# Psychological distress predicts disease activity in inflammatory bowel disease: Results from the mind-body IBD longitudinal study

**DOI:** 10.1016/j.bbih.2025.101147

**Published:** 2025-12-03

**Authors:** Natasha Seaton, Joanna L. Hudson, Valeria Mondelli, Leah Raymond, Pooja Schmill, Ailsa Hart, Rona Moss-Morris

**Affiliations:** a Psychology, Institute of Psychiatry, Psychology and Neuroscience, King's College London, London, UK; b Psychological Medicine, Institute of Psychiatry, Psychology and Neuroscience, King's College London, London, UK; c IBD Unit, St Mark's Hospital, London, UK; d Faculty of Medicine, Imperial College, London, UK

## Abstract

**Background & aims:**

Psychological distress (depression, anxiety, stress) is common in inflammatory bowel disease (IBD) and is linked to poorer outcomes, yet behavioural pathways and economic consequences remain unclear. This study tested a gut-brain-behaviour-outcome (GBBO) framework, testing: 1) reciprocal links between distress and disease activity; 2) whether health behaviours mediate distress-disease activity relationships; 3) if distress and self-reported disease activity (SRDA) predict adverse disease outcomes, controlling for inflammation and 4) whether disease activity mediates relationships between distress and adverse outcomes.

**Methods:**

IBD patients (n = 157) reported distress, health behaviours, SRDA, healthcare use and disease-related outcomes at 3 waves at 6-month intervals. Faecal calprotectin (FCP) was assayed at baseline and 6 months. Analyses were conducted using structural equation modelling and mixed-effects models.

**Results:**

Baseline distress predicted higher SRDA six months later (β = 0.16, *p=.*03) but did not predict FCP; reverse pathways were nonsignificant. Impaired sleep quality mediated 55 % of the effect of distress on future SRDA (β = 0.09, *p=.*04). Other behaviours were nonsignificant. Controlling for FCP, distress and SRDA independently predicted secondary healthcare usage (primary/secondary care) and disease-related outcomes (flare frequency/severity, absenteeism and productivity loss). FCP was positively related to flare frequency/severity and allied health practitioner visits only. Mediation analysis showed that SRDA partially mediated absenteeism.

**Conclusions:**

Psychological distress exacerbates symptoms and economic cost. Poor sleep partially mediates the relationship between distress and SRDA, but not inflammation. Psychological interventions that focus on improving sleep could be cost-effective ways to improve mental health and symptom burden, with downstream impact on healthcare utilisation and productivity losses.

## 1 Introduction

Inflammatory Bowel Disease (IBD), comprising Crohn's disease (CD) and ulcerative colitis (UC), is a chronic relapsing-remitting inflammatory condition of the gastrointestinal tract. Although biologic therapies have lowered rates of surgery and hospitalisation (Moss, 2015), a substantial proportion of patients continue to report debilitating symptoms and impaired quality of life (Follin-Arbelet et al., 2022; Roukas et al., 2024). The cost of care remains significant. In high income countries, annual direct costs are estimated at $12,294 USD for CD and $8782 USD for UC (Burisch et al., 2023), with evidence that the improvements in care conferred by biologics are not offset by reductions in surgeries and other procedures (Burisch et al., 2023; Pillai et al., 2017). Moreover, indirect costs, including productivity losses and workforce absenteeism, compound this burden (Burisch et al., 2024).

Key risk factors for greater healthcare costs and societal impact include active disease, use of steroids and narcotics, low social support, food insecurity, history of smoking and comorbid physical health conditions (Alley et al., 2019; Bähler et al., 2017; Ballou et al., 2018; Ghosh and Premchand, 2015; Janak et al., 2024; Limsrivilai et al., 2017; Nguyen et al., 2021). Among these factors, psychological distress (encompassing chronic stress, depression and anxiety) emerges as a consistent driver of healthcare expenditure and financial burden (Click et al., 2016; Irving et al., 2021; Janak et al., 2024; Sciberras et al., 2022; Szigethy et al., 2021; Wong et al., 2018). These relationships are thought to be underpinned by the gut-brain axis, a multi-faceted bidirectional communication system between the gastrointestinal system and the central nervous system, providing a mechanistic pathway linking psychological factors and gastrointestinal disease activity (Craig et al., 2022; Ge and Liu, 2022; Günther et al., 2021). Animal models have shown that stress alters neuro-visceral integration, immune signalling and microbial composition, thereby potentiating gut inflammation and gastrointestinal symptoms (Ge and Liu, 2022). These findings are reflected in meta-analyses of clinical research suggesting that targeting psychological or microbial pathways can influence symptoms, inflammation and mental health, however, the neuroimmune and microbial pathways that potentially underlie these effects are rarely tested (Kilinçarslan and Evrensel, 2020; Seaton et al., 2024; Tan et al., 2022).

The influence of distress on IBD is not confined to inflammation. Psychological factors can exacerbate self-reported disease activity (SRDA) (Mules et al., 2022), even when objective inflammation is controlled (Binion, 2014; Fairbrass et al., 2020; Sweeney et al., 2018), possibly through altered gut motility and sensitivity (Ge and Liu, 2022; Mayer et al., 2014; Tache et al., 2018; Vanuytsel et al., 2014), or through cognitive-affective processes such as somatic hypervigilance (Crombez et al., 2005; Hughes et al., 2016; Regev et al., 2021), visceral anxiety (Guadagnoli et al., 2023; Wileman et al., 2024), pain catastrophising (Sweeney et al., 2018), and negative illness perceptions (Kantidakis et al., 2021; van der Have et al., 2015). Consistent with this, patient-reported outcome measures (PROMs) for SRDA correlate more strongly with psychological factors than with objective inflammatory biomarkers (Click et al., 2015; Coates and Binion, 2021; Falvey et al., 2015; Gracie et al., 2016; Seaton et al., 2025a). Yet SRDA PROMs are increasingly being used for disease monitoring, treatment decisions and as end-points in clinical trials (Fletcher et al., 2021; Kim et al., 2017; Williet et al., 2014), raising the possibility that untreated distress could inflate symptom scores and illness-related disability (Han and Pae, 2015; Wileman et al., 2024) and consequently increase healthcare and economic burden, irrespective of inflammation. Furthermore, distress may directly impact healthcare usage through increased help-seeking behaviour arising from increased health anxiety or cognitive load of coping with IBD (Gandhi et al., 2014; Seaton et al., 2025b). In a recent cross-sectional study of 599 IBD patients, SRDA and psychological distress each independently predicted healthcare utilisation and economic loss when inflammation was statistically controlled (Seaton et al., 2025a), implying both direct (behavioural) and indirect (symptom-mediated) effects on costs. However, causal inference was precluded by the cross-sectional design, and longitudinal studies are needed to test the temporal ordering of these relationships.

**Fig. 1:**
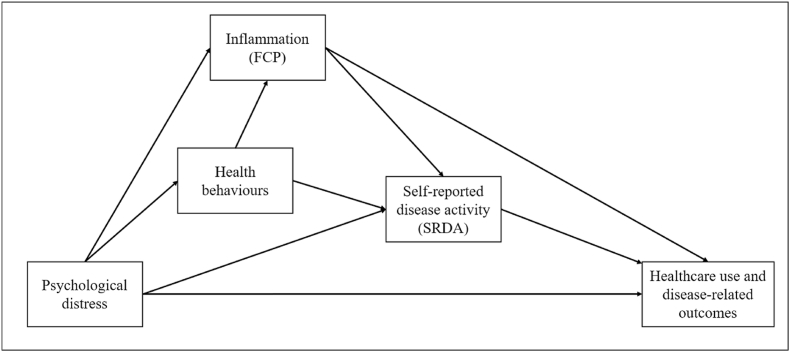
Proposed Gut-Brain-Behaviour-Outcomes (GBBO) Framework through which distress impacts symptomatic and inflammatory disease activity in IBD, and the contribution to secondary healthcare usage and disease-related outcomes.

Given these complex relationships, we propose a Gut-Brain-Behaviour-Outcomes (GBBO) Framework (Fig. 1), with three measurement timepoints (T0 at study start, T1 at 6-months and T2 at 12-months). The GBBO proposes that distress predicts both subsequent SRDA and biochemical disease activity, modelled as related but parallel outcomes, given their low correlation. Thus, the GBBO Framework separates symptom-perception and inflammatory pathways, clarifying distinct mechanisms through which distress may differentially contribute to clinical burden. Moreover, the GBBO Framework anticipates downstream consequences for healthcare use, quality of life, and economic impact, and allows for these effects to be both transmitted indirectly through subjective or objective disease activity, as well as having a direct effect.

A key advantage of the GBBO Framework is the distinction between SRDA, objective disease activity and health-related outcomes. For instance, a meta-analysis of 12 longitudinal studies (n = 9192) found that psychological distress increased the risk of negative disease-related outcomes such as flare, therapy escalation, hospitalisation, emergency department attendance and surgery (Fairbrass et al., 2021). Most included studies relied on administrative data from hospital records, including clinical decisions, which typically consider a broad range of factors and disease indices that are composite of biomarkers, patient reported symptoms and physician assessment (Bitton et al., 2008; Gracie et al., 2018; Kochar et al., 2018; Langhorst et al., 2013; Marrie et al., 2021; Mikocka-Walus et al., 2016). These studies offer unique strengths, by utilising large samples that reflect real-world settings. However, clinical decisions and disease indices that combine patient and/or physician subjective reports and biomarkers, obscure the distinction between inflammatory activity and self-reported symptoms, and their respective contributions to adverse health outcomes. A recent cohort study (n = 110) of UC patients showed that distress predicted clinical flare as measured by a disease activity PROM, but not biochemical flares (Sauk et al., 2023), possibly due to power constraints. Biochemical flares were assessed using faecal calprotectin (FCP), a sensitive marker of inflammation in the gastrointestinal tract (Fagherol et al., 1990), used in IBD for diagnosis and disease monitoring (Pathirana et al., 2018). Larger, longitudinal studies capturing both subjective and objective disease activity are needed to account for the discrepancy between the measures and clarify their respective influence on healthcare and economic costs. Such designs could assess whether distress directly impacts healthcare-economic outcomes, or if the relationships are mediated by increased SRDA and/or inflammation.

Finally, although the gut-brain axis is often cited as a physiological framework to explain direct effects of distress on disease, distress also negatively predicts health behaviours, including diet, sleep, exercise, medication adherence, alcohol consumption and smoking (Bourke et al., 2025; O'Connor et al., 2021). These behaviours independently contribute to health perceptions, inflammation and immune activity (Besedovsky et al., 2012; Galland, 2010; Irwin et al., 2016; Nordgren et al., 2022; O'Connor and Irwin, 2010; Wang et al., 2023), and so are included as important mediators in the GBBO. In IBD, poor sleep quality (Xu et al., 2024), low exercise levels (Jones et al., 2015), low medication adherence (Gohil et al., 2022; Jackson et al., 2010; Kane and Shaya, 2008; Selinger et al., 2011), and higher alcohol intake (Ramos and Kane, 2021) increase the risk of relapse, whereas healthy lifestyle can reduce risk of mortality, flare and disease progression (Lewis et al., 2021; Lo et al., 2021; Rozich et al., 2020). No study has formally tested whether these behaviours mediate the impact of distress on inflammatory or symptomatic disease activity in IBD. Demonstrating mediation would identify concrete behavioural targets that could simultaneously address distress, inflammation, symptom burden, healthcare expenditure and economic impact.

### 1.1 Rationale for study

In summary, distress has wide-ranging health and societal impact in IBD, however, studies are limited in the conflation of symptom-based and biochemical outcomes, leaving it unclear whether distress increases inflammation or primarily heightens symptom perception. Cross-sectional research suggests that distress inflates healthcare utilisation and economic burden independently of inflammation, but temporal precedence and potential mechanisms through SRDA remain unverified. Despite well-documented relationships with distress and disease activity separately, health behaviours have not formally been tested as mediators of the distress-disease relationship.

To address these gaps, we conducted a year-long, cohort study to test the GBBO Framework (Fig. 1). The GBBO proposes that distress predicts subsequent SRDA and biochemical disease activity, partly via health behaviours. We also investigated if these relationships were reciprocal i.e., if FCP and SRDA predict future distress. We also explored whether distress may impact secondary healthcare usage and disease-related outcomes directly, via help-seeking, or indirectly by exacerbating SRDA, when controlling for inflammation.

## 2 Objectives

1. Assess the relationships of psychological distress and disease activity, indexed via two parallel channels: 1) inflammatory disease activity (measured by FCP); and 2) SRDA (measured by a validated PROM). Reciprocal relationships will also be assessed to account for the potential bidirectional influence.
2. Determine whether health behaviours at T1 (sleep, diet, exercise, adherence, smoking) mediate the relationship between psychological distress (T0) and both inflammation (FCP at T1 and SRDA at T2.
3. Evaluate whether psychological distress and SRDA at the prior wave (T_x-1_) prospectively predict healthcare usage and disease-related outcomes (T_x_), when inflammation is controlled (T_x-1_).
4. Quantify the direct effect of T0 distress on T2 secondary healthcare usage and disease-related outcomes, and the indirect effect mediated by disease activity (T1), testing SRDA and FCP as parallel mediators.

## 3 Methods

### 3.1 Design and recruitment

This study was a UK-based online 12-month longitudinal prospective cohort study (Clinical Trials ID: NCT06116331). All study procedures were delivered using secure web-based platforms and postal biological samples.

Inclusion criteria were: (1) self-reporting a clinician diagnosis of CD, UC or IBD-Unclassified (IBD-U); (2) experiencing ≥1 disease flare requiring medical escalation in the preceding 2 years; (3) UK residence and registration with a GP; and (4) ability to complete English-language online surveys. Exclusion criteria were inability to give informed consent, regular non-steroidal anti-inflammatory drug use within the last 2 weeks, or a current cancer diagnosis. Recruitment took place over a 6-month period via social media. Interested individuals underwent a structured screening call, with questions mapped to the above criteria. This included an interview about IBD-related medical and pharmaceutical history (IBD subtype, diagnosis year/location, current consultant managing their IBD care, current medications, and medication history) to help confirm the self-reported diagnosis. If responses indicated that the individual did not have a diagnosis of IBD (e.g., no history of prescribed IBD medication, unable to name hospital/consultant, reported a self-diagnosis), they were excluded. Flow of participants through screening, and reasons for ineligibility, are shown in Supplement 1.

### 3.2 Procedures

Qualtrics survey links were emailed at three timepoints, baseline (T0), 6-months (T1) and 12-months (T2). At-home stool-sampling and mail kits were posted at T0 and T1. Synnovis Laboratories (King's College Hospital, London) analysed samples for calprotectin concentration (μg/g) using the Diasorin Liaison XL Calprotectin assay, an in vitro diagnostic chemiluminescent immunoassay (CLIA).

Informed consent was obtained from all participants for this study. Ethical approval was obtained from King's College London Research Ethics Committee (HR/DP-22/23–33844). All procedures were conducted in accordance with the Declaration of Helsinki.

### 3.3 Measures

Fig. 1 shows the categories of key measures in this study. Psychological distress was the key predictor variable, and objective and subjective disease activity were the primary outcomes. Health behaviours were included as mediators, and additional secondary outcomes including healthcare usage and disease-related outcomes. Objective disease activity was assessed with FCP (T0, T1). All other data were collected in self-reported questionnaires at 3 timepoints (T0, T1, T2).

#### 3.3.1 Key variables

Psychological distress was captured with the Patient Health Questionnaire-Anxiety and Depression Scale (PHQ-ADS) (Kroenke et al., 2016), which is a composite of the 9-item Patient Health Questionnaire (PHQ-9) (Kroenke and Spitzer, 2002) for depression and the 7-item Generalised Anxiety Disorder Scale (GAD-7) for anxiety (Spitzer et al., 2006) (range: 0–48, higher scores indicate more distress, α = .92–0.93).

Self-reported disease activity (SRDA) was assessed with PROMs that are valid and reliable in IBD research (Jairath et al., 2015; Khanna et al., 2015). Displayed questions depended on disease subtype and presence of stoma. Questions pertained to abdominal pain severity over the last 7 days (all), stool frequency over the last 7 days (all except stoma), and rectal bleeding over the last 3 days (UC and no stoma only). A z score was calculated for inferential statistics in the whole sample.

Objective intestinal inflammation was measured with faecal calprotectin (FCP), a sensitive marker of inflammation in the gastrointestinal tract (Fagherol et al., 1990), used in IBD for diagnosis and disease monitoring (Pathirana et al., 2018). Levels <100 μg/g reflect minimal inflammation; ≥100 and < 250 μg/g represents possible inflammation and ≥250 μg/g indicates active inflammation (Bressler et al., 2015). FCP was still collected for ileostomy patients, given high sensitivity and specificity for detecting small bowel inflammation (Daoud et al., 2022; Park et al., 2025).

#### 3.3.2 Mediator variables

Exercise was measured with the International Physical Activity Questionnaire (IPAQ) (Craig et al., 2003) where participants self-report vigorous, moderate, and lower-level activities (walking). Metabolic Equivalent Task (MET) values are used to compute an overall score (walking = 3.3., moderate = 4.0 and vigorous = 8.0) (Lee et al., 2011).

Diet was evaluated with an 8-item healthy eating assessment (Paxton et al., 2011; Thompson et al., 2004) designed for use in primary care settings (range: 8–40), higher scores indicate better diet, α = .35–0.39).

Adherence was measured using the 5-item Medication Adherence Report Scale (MARS) (Chan et al., 2020). Higher scores indicate higher adherence rates (range:5–25, α = .76–0.79).

Sleep was assessed with the Pittsburgh Sleep Quality Index (PSQI; Buysse et al., 1989), which measures sleep quality in the past month (range:0–21). Higher scores indicate worse sleep quality (α = .72–0.75).

Additional questions for smoking behaviour were asked.

#### 3.3.3 Secondary outcomes (healthcare use and disease-related outcomes)

Healthcare usage was assessed using an adapted IBD Resource Use Questionnaire (Roukas et al., 2023), asking participants the frequency of use for various health services in the last six months. These included GP visits, gastroenterologist, colorectal surgeon, pharmacist, IBD nurse, IBD advice line, psychologist, dietician, Accident and Emergency (A&E) and hospitalisations. For this study, relevant service use was summed to compute composite scores for primary care (GP and pharmacist), secondary care (gastroenterologist, colorectal surgeon, IBD nurse and IBD advice line) and for allied health professionals (psychologist and dietician).

IBD-related quality of life was assessed with the 10-item short Inflammatory Bowel Disease Questionnaire (sIBDQ) (range: 10–70, higher scores better quality of life, α = .90–0.91) (Irvine et al., 1996).

Additional secondary outcomes asked about the number of self-reported flares, severity of previous flare, absenteeism (number of sick-leave days due to IBD), and impact of IBD on productivity (0 = no effect on work, 10 = completely prevented me from working) over the last 6 months.

#### 3.3.4 Demographic and other illness-related variables

The baseline questionnaire collected demographic (age, gender, ethnicity, education level, employment, and postcode for socioeconomic status calculation) and clinical information (IBD subtype, comorbidities and diagnosis length). See Supplement 2 for information on covariate coding. Presence of stoma, recent operations, height and weight (for BMI calculations) and IBD medications were collected at all timepoints.

### 3.4 Sample size

Based on prior literature (Fairbrass et al., 2021) indicating a small-to-moderate association between distress and subsequent adverse IBD outcomes (RR:1.3–1.6), 108 participants would provide 80 % power (α = .05, four predictors). Allowing for attrition and additional exploratory analysis, the target sample was 150.

### 3.5 Statistical analysis

**Fig. 2:**
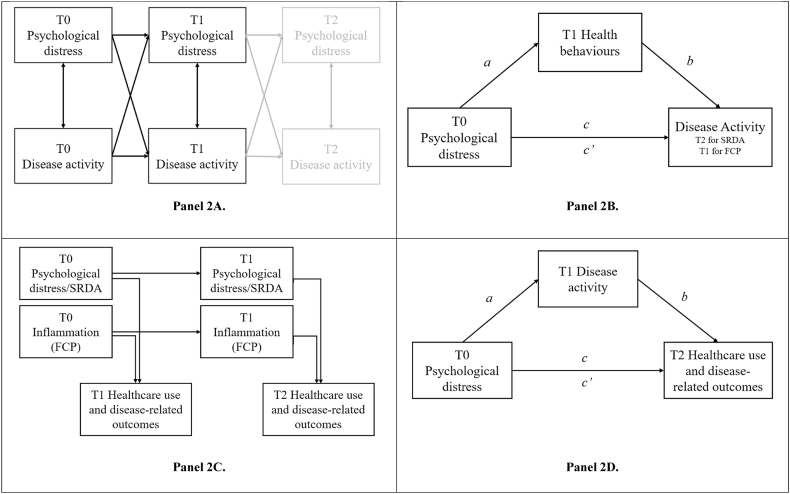
Structural equational modelling and mixed-effects models used to test the Gut-Brain-Behaviour-Outcomes (GBBO) framework.

Analyses were conducted with STATA v18. Bivariate analyses (e.g., independent sample t-tests, χ^2^ tests and Fisher's exact tests) were run to assess differences between dropouts and retained participants. Regression models of demographic variables with SRDA and FCP were used to determine relevant sociodemographic and clinical confounders. Given the skew commonly observed in biomarkers (Curran-Everett, 2018), FCP was log-transformed prior to analysis. To make the time ordering explicit, we label the three waves as T0 (baseline), T1 (6 months) and T2 (12 months). Fig. 2 presents the tested models for each objective. For the first objective, the reciprocal relationships for disease activity and distress were investigated with cross-lagged panel models (CLPMs), fitted in structural equational modelling (SEM) frameworks (FCP: T0, T1; SRDA: T0, T1, T2). This assessed whether earlier distress predicts later disease activity and vice versa.

For the second objective, mediation was assessed using SEM. For SRDA, T0 distress was the independent variable, T2 SRDA was the outcome variable, and the T1 health behaviour was used as the mediator. As FCP was only collected at two timepoints, T1 FCP was the outcome variable. Mediation models controlled for baseline measures of the mediator and/or outcome as confounders. These were likely to provide adjustment for other related confounders not included in the model. SEM analyses used maximum likelihood estimation to handle missing data to minimise bias and conserve sensitivity of the analysis (Enders and Bandalos, 2001).

For the third objective, mixed-effects models with random intercepts were used to account for repeated measures. These assessed the influence of distress/SRDA on the secondary outcomes whilst controlling for inflammation. Time-lagged predictors from the prior wave (T_x-1_; distress for mixed-model 1, SRDA for mixed-model 2, FCP in both models) alongside relevant covariates were used to predict the healthcare use and disease-related outcomes at the next wave 6-months later (T_x_). The notations T_x-1_ for predictors and T_x_ for outcomes indicate that predictors collected in previous wave 6-months earlier are inputted, thus modelling T0 →T1 and T1 →T2 effects concurrently. Linear, logistic or negative binomial mixed-effects models were used for continuous, binary and count outcomes, respectively. Random intercepts handled within-person repeated assessments. Analyses controlled for baseline values of outcomes.

The fourth objective tested if disease activity (both SRDA and FCP) mediated the relationship between distress and secondary outcomes, using SEM (generalised SEM for non-parametric outcomes, such as count or binary outcomes), with baseline values for outcome and mediator included as controls. FCP was additionally controlled when SRDA was tested as a mediator. For all estimates, SE, p and 95 % CIs were reported, alongside standardised betas, odds ratios and incident rate ratios (IRR) for linear, logistic and negative binomial analyses, respectively. All SEM and mixed-effects model analyses used maximum likelihood estimation to handle missing data to minimise bias and conserve sensitivity of the analysis (Enders and Bandalos, 2001).

## 4 Results

Of 157 adults enrolled between May 2023 and January 2024, 142 (90.4 %) completed the 6-month assessment (T1) and 139 (88.5 %) completed the 12-month assessment (T2) (Participant flow in Supplement 1); full-information maximum-likelihood estimation allowed all 157 participants to contribute to longitudinal models. The cohort was predominantly female (73.9 %) and white (82.2 %). Over half (58.0 %) were in biochemical remission (FCP<100 μg/g), yet mean psychological distress was in the mild-to-moderate range (M = 17.3, SD = 10.4). Table 1 presents full demographic and clinical data. Participants who did not provide follow-up data were compared with those retained in the study. There were no significant differences for most demographics, psychological factors or faecal calprotectin; however, dropouts had higher SRDA and lower education level at baseline (See Supplement 3). Relationships of demographic and clinical variables with distress, SRDA and FCP are in Supplement 4.

**Table 1:** Demographic and clinical information for participants at baseline.

Demographic characteristics		n (%)	M (SD)
Age (min = 18, max = 76)		–	35.85 (11.58)
Gender			
	Male	41 (25.95 %)	–
	Female	116 (73.89 %)	–
Ethnicity			
	White	129 (82.17 %)	–
	Mixed/multiple ethnic groups	6 (3.82 %)	–
	Asian or Asian British	16 (10.19 %)	–
	Black or Black British	2 (1.27 %)	–
	Arab	4 (2.55 %)	–
Index of Multiple Deprivation Decile^$^ (min = 0.0, max = 1.0)		–	0.54 (0.28)
Employment status			
	Employed	129 (82.17 %)	–
	Unemployed	12 (7.64 %)	–
	Retired	10 (6.37 %)	–
	Student	6 (3.82 %)	–
Education level			
	None	1 (0.64 %)	–
	School	33 (21.02 %)	–
	Undergraduate	70 (44.59 %)	–
	Postgraduate	53 (33.76 %)	–
Clinical characteristics		n (%)	M(SD)
Diagnosis			
	Crohn's Disease	52 (33.12 %)	–
	Ulcerative Colitis	93 (59.24 %)	–
	Indeterminate	10 (6.37 %)	–
	Unsure	2 (1.27 %)	–
Years since diagnosis (min = 0, max = 40)		–	7.90 (8.25)
Medications			
	Aminosalicylates/5-ASA	77 (49.04 %)	–
	Immunomodulators	40 (25.48 %)	–
	Biologics	77 (49.04 %)	–
	Steroids	29 (18.47 %)	–
	No IBD medication	18 (11.46 %)	–
Body Mass Index			
	Overweight	58 (36.94 %)	
	Healthy weight	87 (55.41 %)	
	Underweight	8 (5.10 %)	
	Missing	4 (2.55 %)	
Stoma			
	Yes	9 (5.73 %)	
	No	148 (94.27 %)	
**Faecal Calprotectin** (median = 54, IQR = 14–199)			309.11 (881.59)
	Remission	91 (57.96 %)	
	Borderline	18 (11.46 %)	
	Active disease	36 (22.93 %)	
	Missing	12 (7.64 %)	
Anxiety (GAD-7)			8.59 (5.45)
Depression (PHQ-9)			8.73 (4.84)
**Distress** (PHQ-ADS)			17.32 (10.38)
Disease activity PROM (CD) (n = 46, min = 0, max = 7.3)			2.34 (1.74)
Disease activity PROM (UC) (n = 98, min = 0, max = 9.0)			2.12 (2.06)
Disease activity PROM (stoma) (n = 9, min = 0, max = 2.1)			0.81 (0.87)

$deprivation was computed using postcodes in national registries, deprivation data were missing for 3 postcodes. CD=Crohn's Disease, GAD-7 = Generalised Anxiety Disorder Scale, IQR = interquartile range, PHQ-9 Patient Health Questionnaire, PHQ-ADS=Patient Health Questionnaire Anxiety and Depression Scale, PROM = patient reported outcome measure, UC = ulcerative colitis.

### 4.1 Objective 1: reciprocal relationships between psychological distress and disease activity

**Fig. 3:**
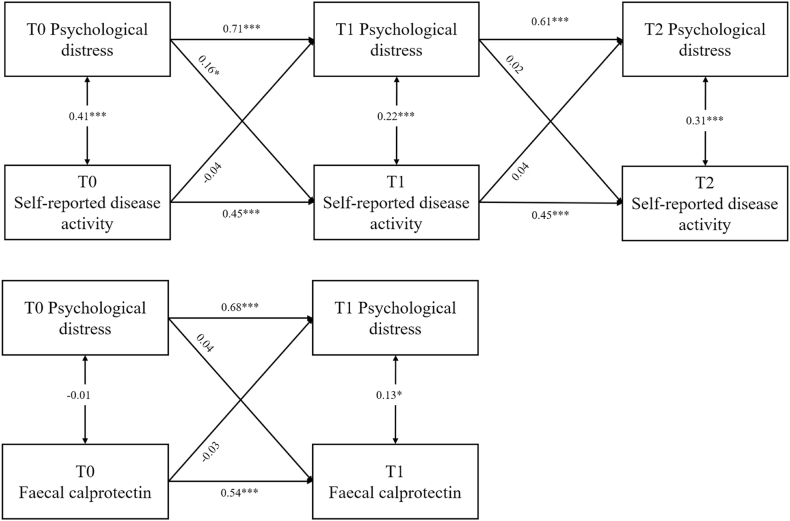
Cross-lagged panel model (CLPM) results for psychological distress with both self-reported disease activity and objective inflammation.

**Self-reported disease activity (SRDA, T0-T1-T2).** The CLPMs (Fig. 2A) showed that baseline (T0) distress predicted higher SRDA six months (T1) later (β = 0.159, *p* = .030, 95 %CIs: 0.02, 0.30), after adjusting for baseline SRDA (β = 0.448, p < .001, 95 %CIs: 0.30, 0.60). After adjusting for T1 SRDA, T1 distress did not significantly predict T2 SRDA (β = 0.023, p = .797, 95 %CI: 0.15, 0.20); however, the confidence intervals from the T0-T1 and T1-T2 predictions overlapped, showing no significant differences in estimate. Cross-sectional correlations between distress and SRDA ranged from small to moderate at every wave (β = 0.22–0.41; all p < .001). The CLPM diagram is shown in Fig. 3, with full results in Supplement 5.

**Inflammatory activity (FCP, T0-T1).** Neither direction of the distress–FCP pathway reached significance longitudinally. Baseline distress did not predict FCP at 6 months (β = −0.037, p = .610). Cross-sectionally, distress and FCP were modestly, cross-sectionally associated at T1 (β = 0.129, p = .019) but not T0 (β = −0.007, p = .934). The CLPM diagram is shown in Fig. 3, with full results in Supplement 6.

### 4.2 Objective 2: health behaviour (T1) as a mediator of distress (T0) and disease activity (T2)

Table 2 shows results from the health behaviour SEMs (Fig. 2B). For SRDA, there was a small significant overall effect of baseline (T0) distress predicting worse SRDA 12-months (T2) later (β = 0.180, SE = 0.07, p = .028, 95 %CIs: 0.02, 0.34), controlling for baseline SRDA (T0). Sleep quality (T1) significantly mediated the effect of baseline distress on T2 SRDA (indirect path: β = 0.093, p = .044, 95 %CIs: 0.00, 0.18), accounting for 55.5 % (95 %CIs: 47.3 %, 63.3 %) of the total effect. T0 distress moderately predicted poorer sleep at 6 months (T1) (β = 0.475, p < .001, 95 %CIs: 0.31, 0.64), and poorer sleep in turn predicted higher T2 SRDA (β = 0.120, p = .031, 95 %CIs: 0.02, 0.37). Baseline distress predicted poorer diet and exercise at T1 (|β| = 0.282–0.299, both p < .01), but these health behaviours did not significantly predict T2 SRDA, yielding no significant mediation.

**Table 2:** Total, direct, indirect effects and mediation paths for all models.

			Self-reported disease activity			Faecal Calprotectin		
			β (SE)	p	95 %CIs	β (SE)	p	95 %CIs
Total effect	Distress→DA		0.180 (0.08)	0.028	0.02, 0.34	0.016 (0.07)	0.825	−0.13, 0.16

Sleep								
	Path a	Distress→Sleep	0.475 (0.08)	<0.001	0.31, 0.64	0.071 (0.08)	0.349	−0.08, 0.22
	Path b	Sleep→DA	0.120 (0.09)	0.031	0.02, 0.37	0.191 (0.11)	0.074	−0.02, 0.40
	Indirect	Distress→Sleep→ DA	0.093 (0.05)	0.044	0.00, 0.18	0.014 (0.02)	0.407	−0.02, 0.05
	Direct	Distress→DA∗	0.075 (0.09)	0.427	−0.11, 0.26	0.109 (0.09)	0.238	−0.07, 0.29
Diet								
	Path a	Distress→Diet	−0.299 (0.10)	0.002	−0.49, −0.11	−0.150 (0.07)	0.041	−0.29, −0.01
	Path b	Diet → DA	−0.060 (0.08)	0.459	−0.22, 0.10	−0.151 (0.09)	0.089	−0.33, 0.02
	Indirect	Distress→ Diet → DA	0.018 (0.02)	0.473	−0.03, 0.07	0.023 (0.02)	0.191	−0.01, 0.06
	Direct	Distress→DA∗	0.164 (0.08)	0.052	−0.00, 0.33	−0.019 (0.08)	0.798	−0.17, 0.13
Exercise								
	Path a	Distress→ Exercise	−0.282 (0.08)	0.001	−0.45, −0.12	−0.185 (0.08)	0.012	−0.33, −0.04
	Path b	Exercise → DA	0.109 (0.09)	0.231	−0.07, 0.29	−0.047 (0.09)	0.589	−0.22, 0.12
	Indirect	Distress→Exercise→DA	−0.031 (0.03)	0.258	−0.08, 0.02	0.009 (0.01)	0.597	−0.02, 0.04
	Direct	Distress→DA∗	0.211 (0.09)	0.013	0.04, 0.38	0.040 (0.08)	0.600	−0.11, 0.19
Adherence								
	Path a	Distress→ Adherence	−0.067 (0.09)	0.475	−0.25, 0.12	−0.017 (0.07)	0.800	−0.16, 0.12
	Path b	Adherence → DA	0.053 (0.08)	0.514	−0.11, 0.21	−0.012 (0.09)	0.894	−0.20, 0.17
	Indirect	Distress→Adherence→DA	−0.004 (0.01)	0.628	−0.02, 0.01	0.000 (0.00)	0.906	−0.00, 0.00
	Direct	Distress→DA∗	0.185 (0.08)	0.024	0.02, 0.35	−0.027 (0.07)	0.715	−0.17, 0.12
Smoking								
	Path a	Distress→ Smoking	0.122 (0.12)	0.320	−0.12, 0.36	0.095 (0.11)	0.394	−0.12, 0.31
	Path b	Smoking → DA	−0.007 (0.06)	0.904	−0.13, 0.11	0.046 (0.06)	0.428	−0.07, 0.16
	Indirect	Distress→ Smoking→ DA	−0.001 (0.01)	0.904	−0.02, 0.01	0.004 (0.01)	0.561	−0.01, 0.02
	Direct	Distress→DA∗	0.181 (0.08)	0.027	0.02, 0.34	0.012 (0.07)	0.865	−0.13, 0.16

*Note*. DA = disease activity.

There was no significant relationship between T0 distress and T1 disease activity as measured by FCP (β = 0.016 (0.07), p = .825, 95 %CIs: 0.13, 0.16); however, paths were still investigated to explore if health behaviours were related to inflammatory disease activity. Impaired sleep (β = 0.191, p = .074, 95 %CIs: 0.02, 0.40) and poorer diet (β = −0.15, p = .089, 95 %CIs: 0.33, 0.02) showed nonsignificant trends with levels of FCP at T1. Medication adherence and smoking status were not associated with distress or either measure of disease activity (SRDA and FCP).

Sensitivity analyses were re-run controlling for age, gender, obesity, biologics use and steroids use, demonstrating no meaningful change in size or significance of estimates (See Supplement 7). Interestingly, obesity was significantly associated with worsened SRDA 12-months later (β = 0.335, p = .050, 95 %CIs: 0.00, 0.67), but not FCP. Other covariates were nonsignificant.

### 4.3 Objective 3: distress and SRDA (Tx-1) as longitudinal predictors of secondary healthcare use and disease-related outcomes (Tx)

Mixed-effects models controlling for prior FCP (Fig. 2C) are summarised in Table 3.

**Table 3:** Mixed-effects models of psychological distress or SRDA predicting secondary healthcare usage and disease-related outcomes, statistically controlling for baseline values and FCP.

		Faecal Calprotectin			Psychological Distress			Self-reported disease activity		
		IRR/β/OR	p	95 %CIs	IRR/β/OR	p	95 %CIs	IRR/β/OR	p	95 %CIs
Flares total (IRR)										
	Mixed-model 1	1.406 (0.14)	0.001	1.15, 1.71	1.279 (0.13)	0.014	1.05, 1.56			
	Mixed-model 2	1.291 (0.12)	0.008	1.07, 1.56				1.488 (0.13)	<0.001	1.25, 1.77
Flare severity (OR)										
	Mixed-model 1	2.546 (0.59)	<0.001	1.62, 4.00	1.727 (0.38)	0.014	1.12, 2.67			
	Mixed-model 2	2.200 (0.48)	<0.001	1.44, 3.37				2.345 (0.54)	<0.001	1.49, 3.69
Primary visits (IRR)										
	Mixed-model 1	0.937 (0.05)	0.237	0.84, 1.04	1.108 (0.06)	0.050	1.00, 1.23			
	Mixed-model 2	0.896 (0.05)	0.062	0.80, 1.01				1.106 (0.06)	0.067	0.99, 1.23
Secondary visits (IRR)										
	Mixed-model 1	1.095 (0.07)	0.144	0.97, 1.24	1.070 (0.07)	0.303	0.94, 1.22			
	Mixed-model 2	1.050 (0.07)	0.444	0.93, 1.19				1.177 (0.07)	0.008	1.04, 1.33
Allied visits (IRR)										
	Mixed-model 1	1.253 (0.13)	0.028	1.02, 1.53	1.161 (0.11)	0.122	0.96, 1.40			
	Mixed-model 2	1.230 (0.13)	0.046	1.00, 1.51				1.011 (0.10)	0.910	0.84,1.22
Absenteeism (IRR)										
	Mixed-model 1	1.005 (0.11)	0.961	0.81, 1.25	1.828 (0.22)	<0.001	1.45, 2.30			
	Mixed-model 2	0.939 (0.11)	0.585	0.75, 1.18				1.490 (0.18)	0.001	1.17, 1.90
Hospitalisations (IRR)										
	Mixed-model 1	1.440 (0.37)	0.160	0.87, 2.40	1.556 (0.38)	0.067	0.97, 2.50			
	Mixed-model 2	1.241 (0.35)	0.442	0.72, 2.15				1.615 (0.38)	0.044	1.01, 2.57
Productivity impact (β)										
	Mixed-model 1	0.052 (0.06)	0.367	−0.06, 0.17	0.225 (0.07)	0.001	0.10, 0.35			
	Mixed-model 2	0.043 (0.06)	0.488	−0.08, 0.16				0.119 (0.07)	0.079	−0.01, 0.25
IBD-QoL (β)										
	Mixed-model 1	0.044 (0.04)	0.286	−0.04, 0.12	−0.126 (0.05)	0.018	−0.23, −0.02			
	Mixed-model 2	0.066 (0.04)	0.129	−0.02, 0.15				−0.101 (0.05)	0.045	−0.20, −0.00
A&E visit (OR)										
	Mixed-model 1	1.364 (0.39)	0.279	0.78, 2.39	0.994 (0.03)	0.821	0.94, 1.05			
	Mixed-model 2	1.172 (0.34)	0.578	0.67, 2.05				1.251 (0.34)	0.414	0.73, 2.14

*Note*. A&E = accident and emergency, FCP = faecal calprotectin IBD = inflammatory bowel disease, IRR = incident risk ratio, OR = odds ratio. QoL = quality of life.

**Psychological distress (Mixed-model 1: Distress and FCP (T0 and T1, modelled as T_x-1_) predicting secondary outcomes (T1 and T2, modelled as T_x_)).** When controlling for objective inflammation (FCP), distress (T_x-1_) prospectively predicted a variety of secondary outcomes (T_x_) including healthcare utilisation, absenteeism, productivity and quality of life (Table 3). Both distress and FCP (T_x-1_) were significant predictors of future (T_x_) self-reported flare frequency (Distress: IRR = 1.279, p = .014, 95 %CIs: 1.05, 1.56; FCP: IRR = 1.406, p = .001, 95 %CIs: 1.15, 1.71) and flare severity (Distress: OR = 1.727, p = .014, 95 %CIs: 1.12, 2.67; FCP: OR = 2.546, p < .001, 95 %CIs: 1.62, 4.00). Additionally, T_x-1_ distress significantly predicted future (T_x_) primary care contacts (IRR = 1.108, p = .050, 95 %CIs: 1.00, 1.23), absenteeism (IRR = 1.828, p < .001, 95 %CIs: 1.45, 2.30), productivity loss (β = 0.225, p = .001, 95 %CIs: 0.10, 0.35) and IBD-related quality of life (β = −0.126, p = .021, 95 %CIs −0.23, −0.02). There was a nonsignificant trend for hospitalisations (IRR = 1.556, p = .067, 95 %CIs: 0.97, 2.50). Neither distress nor FCP prospectively predicted secondary care visits or IBD-related A&E attendance.

**Self-reported disease activity (Mixed-model 2: SRDA and FCP (T0 and T1, modelled as T_x-1_) predicting secondary outcomes (T1 and T2, modelled as T_x_)).** When controlling for FCP, T_x-1_ SRDA was prospectively associated with certain secondary outcomes. Both T_x-1_ FCP and SRDA were associated with higher number (FCP: IRR = 1.291, p = .008, 95 %CIs: 1.07, 1.56; SRDA: IRR = 1.488, p < .001, 95 %CIs: 1.25, 1.77) and severity (FCP: OR = 2.546, p < .001, 95 %CIs: 1.62, 4.00; SRDA: OR = 2.345, p < .001, 95 %CIs: 1.49, 3.69) of future flares (T_x_). Moreover, T_x-1_ SRDA emerged as significant predictor of T_x_ secondary-care visits (IRR = 1.177, p = .008, 95 %CIs: 1.04, 1.33), absenteeism (IRR = 1.490, p = .001, 95 %CIs: 1.17, 1.90), hospitalisations (IRR = 1.615, p = .044, 95 %CIs: 1.01, 2.57) and worse IBD-related quality of life (β = −0.101, p = .045, 95 %CIs −0.20, −0.00). Neither SRDA nor FCP significantly predicted future A&E or primary-care visits.

For both mixed-model 1 and mixed-model 2, sensitivity analyses were conducted including certain confounders (age, gender, BMI, biologics, steroids, see Supplement 8). Results were largely unchanged, with sensitivity estimates falling within confidence intervals of original analyses.

### 4.4 Objective 4: disease activity (T1) as a mediator of the relationship of distress (T0) and secondary outcomes (T2)

Table 4 shows results of the analysis assessing if T1 disease activity mediates the relationship between T0 distress and secondary healthcare use and disease-related outcomes at T2 (Fig. 2D). Although T0 distress predicted T1 SRDA but not T1 FCP (path a), the mediation analysis was conducted for both metrics of disease activity, so that relationships between T1 FCP and T2 secondary outcomes were still explored (path b).

**Table 4:** Total, direct, indirect effects of models testing self-reported disease activity as a mediator of the effect of distress on healthcare use and disease-related outcomes.

			Self-reported disease activity			Faecal Calprotectin		
			β/IRR/OR (SE)	p	95 %CIs	β/IRR/OR (SE)	p	95 %CIs
Path a	Distress→DA		0.166 (0.07)	0.016	0.03, 0.30	0.016	0.825	−0.13, 0.16

Total flares (IRR)								
	Total effect	Distress→total flares	0.989 (0.16)	0.949	0.71, 1.37	1.064 (0.15)	0.656	0.81, 1.40
	Path b	DA→total flares	1.986 (0.36)	<0.001	1.39, 2.83	1.198 (0.21)	0.292	0.86, 1.68
	Indirect effect	Distress→DA→total flares	1.120 (0.07)	0.064	0.99, 1.26	1.003 (0.01)	0.827	0.98, 1.03
	Direct effect	Distress→total flares∗	0.883 (0.15)	0.461	0.63, 1.23	1.061 (0.15)	0.668	0.81, 1.39
Flare severity (OR)								
	Total effect	Distress→flare severity	1.564 (0.39)	0.070	0.96, 2.53	1.771 (0.36)	0.005	1.18, 2.65
	Path b	DA→flare severity	2.613 (0.61)	<0.001	1.65, 4.14	1.754 (0.40)	0.013	1.12, 2.74
	Indirect effect	Distress→DA→flare severity	1.172 (0.10)	0.065	0.99, 1.39	1.009 (0.04)	0.820	0.93, 1.09
	Direct effect	Distress→flare severity∗	1.334 (0.34)	0.259	0.81, 2.20	1.754 (0.35)	0.005	1.19, 2.59
Primary visits (IRR)								
	Total effect	Distress→primary visits	1.173 (0.10)	0.071	0.99, 1.39	1.200 (0.09)	0.021	1.03, 1.40
	Path b	DA→primary visits	1.071 (0.09)	0.391	0.92, 1.25	1.058 (0.09)	0.488	0.90, 1.24
	Indirect effect	Distress→DA→primary visits	1.011 (0.01)	0.413	0.98, 1.04	1.001 (0.00)	0.825	0.99, 1.01
	Direct effect	Distress→primary visits∗	1.160 (0.11)	0.106	0.97, 1.39	1.199 (0.09)	0.021	1.03, 1.40
Secondary visits (IRR)								
	Total effect	Distress→secondary visits	1.056 (0.12)	0.644	0.84, 1.33	1.078 (0.11)	0.472	0.88, 1.32
	Path b	DA→secondary visits	1.803 (0.28)	<0.001	1.33, 2.45	1.696 (0.31)	0.003	1.19, 2.41
	Indirect effect	Distress→DA→ secondary visits	1.102 (0.06)	0.052	1.00, 1.22	1.009 (0.04)	0.819	0.94, 1.09
	Direct effect	Distress →secondary visits∗	0.958 (0.11)	0.701	0.77, 1.19	1.069 (0.11)	0.501	0.88, 1.30
Allied visits (IRR)								
	Total effect	Distress→allied visits	1.515 (0.33)	0.054	0.99, 2.31	1.429 (0.28)	0.069	0.97, 2.10
	Path b	DA→allied visits	1.075 (0.21)	0.711	0.74, 1.57	0.979 (0.22)	0.926	0.63, 1.52
	Indirect effect	Distress→ DA → allied visits	1.012 (0.03)	0.714	0.95, 1.08	1.000 (0.00)	0.927	0.99, 1.00
	Direct effect	Distress → allied visits∗	1.497 (0.32)	0.056	0.99, 2.26	1.429 (0.28)	0.069	0.97, 2.10
Absenteeism (IRR)								
	Total effect	Distress- > absenteeism	2.330 (0.64)	0.002	1.36, 3.99	2.054 (0.44)	0.001	1.35, 3.12
	Path b	DA→absenteeism	2.340 (0.48)	<0.001	1.56, 3.20	2.898 (0.83)	<0.001	1.65, 5.01
	Indirect effect	Distress→DA→ absenteeism	1.151 (0.08)	0.054	1.00, 1.33	1.018 (0.08)	0.818	0.88, 1.18
	Direct effect	Distress→ absenteeism	2.024 (0.56)	0.010	1.18, 3.47	2.019 (0.43)	<0.001	1.34, 3.01
Hospitalisation (IRR)								
	Total effect	Distress→ hospitalisations	1.562 (0.96)	0.470	0.47, 5.23	1.390 (0.58)	0.431	0.61, 3.15
	Path b	DA→hospitalisations	3.131 (1.77)	0.043	1.04, 9.46	5.441 (1.70)	<0.001	2.95, 10.04
	Indirect effect	Distress→DA→ hospitalisations	1.208 (0.18)	0.198	0.91, 1.61	1.028 (0.12)	0.817	0.81, 1.30
	Direct effect	Distress → hospitalisations ∗	1.294 (0.90)	0.712	0.33, 5.07	1.352 (0.53)	0.440	0.63, 2.91
Productivity impact (β)								
	Total effect	Distress→ productivity impact	0.198 (0.19)	0.035	0.01, 0.38	0.219 (0.09)	0.013	0.05, 0.39
	Path b	DA → productivity impact	0.115 (0.11)	0.290	−0.10, 0.33	0.100 (0.10)	0.310	−0.09, 0.29
	Indirect effect	Distress→ DA → productivity impact	0.019 (0.02)	0.325	−0.02, 0.06	0.003 (0.01)	0.676	−0.01, 0.02
	Direct effect	Distress → productivity impact ∗	0.179 (0.09)	0.054	0.00, 0.36	0.216 (0.09)	0.014	0.04, 0.39
IBD-related QoL (β)								
	Total effect	Distress→ IBD-related QoL	−0.314 (0.06)	<0.001	−0.44, −0.18	−0.342 (0.06)	<0.001	−0.48, −0.24
	Path b	DA → IBD-related QoL	−0.155 (0.07)	0.039	−0.30, −0.01	−0.056 (0.08)	0.470	−0.21, 0.10
	Indirect effect	Distress→ DA → IBD-related QoL	−0.026 (0.02)	0.111	−0.06, 0.01	−0.002 (0.00)	0.676	−0.01, 0.01
	Direct effect	Distress → IBD-related QoL ∗	−0.288 (0.07)	<0.001	−0.42, −0.16	−0.341 (0.06)	<0.001	−0.46, −0.22
A&E visit (OR)								
	Total effect	Distress→ A&E visit	1.171 (0.37)	0.621	0.63, 2.19	1.073 (0.29)	0.793	0.643, 1.81
	Path b	DA → A&E visit	1.641 (0.56)	0.147	0.84, 3.20	1.529 (0.41)	0.114	0.90, 2.59
	Indirect effect	Distress→ DA → A&E visit	1.079 (0.35)	0.816	0.57, 2.04	1.065 (0.28)	0.809	0.64, 1.78
	Direct effect	Distress → A&E visit∗	1.085 (0.08)	0.261	0.94, 1.25	1.007 (0.03)	0.820	0.95, 1.07

*Note*. ∗ indicates where the estimate refers to the direct effect of distress, without the SRDA mediating pathway. All analyses were controlled for faecal calprotectin (FCP), and the baseline of the outcome and the mediator. A&E = accident and emergency, IBD = inflammatory bowel disease, QoL = quality of life, SRDA = self-reported disease activity.

SRDA (T1) significantly partially mediated the effect of distress (T0) on absenteeism (T2). A 1-SD increase in distress directly increased the rate of sick days by 133 % (IRR = 2.330, p = .002) with 17 % of the effect transmitted through changes in SRDA from T0 to T1. For the same 1-SD increase in distress, this corresponds to a 15 % increased rate in sick days (IRR = 1.151, p = .054). Despite T0 distress significantly predicting T2 impact of IBD on productivity (β = 0.198, p = .035), quality of life (β = −0.314, p < .001), allied health visits (IRR = 1.515, p = .054), and showing a nonsignificant trend with primary visits (IRR = 1.173, p = .071), there was no evidence that these effects were mediated through SRDA (|β|<.03 or IRR<1.012, p > .10).

Though T1 SRDA significantly predicted T2 flares (IRR = 1.986, p < .001), secondary care visits (IRR = 1.803, p < .001) and hospitalisations (IRR = 3.131, p = .043), there was no total effect of T0 distress (c path) for these outcomes. For flare severity, there were nonsignificant trends for T0 distress predicting T2 flare severity (IRR = 1.564, p = .07), and the effect being mediated through SRDA (IRR = 1.172, p = .065).

Controlling for baseline of the outcome, T1 FCP significantly predicted flare severity (OR = 1.754, p = .013), secondary visits (IRR = 1.696, p = .003), absenteeism (IRR = 2.898, p < .001) and hospitalisation (IRR = 5.441, p < .001), but was not related to other outcomes. Table 4 shows full results.

## 5 Discussion

This 12-month prospective cohort study of 157 adults with IBD tested a GBBO framework (Fig. 1), investigating relationships between psychological distress, disease activity and adverse IBD outcomes, while including health behaviours as potential mediators. Cross-lagged modelling showed that baseline psychological distress predicted SRDA 6-months later, but there was no significant relationship with FCP. Reciprocal pathways of disease activity predicting the emergence of distress were nonsignificant. Sleep quality emerged as a statistically significant mediator of the distress-SRDA pathway, accounting for more than half of the total effect. As there was no total effect for distress-FCP, mediation could not be assessed; however, there were nonsignificant trends suggesting impaired sleep quality and poor diet were related to higher inflammation. Medication adherence, exercise and smoking did not mediate any distress-disease links.

When inflammation was statistically controlled, distress and SRDA independently predicted many of the healthcare use and disease-related outcomes measured, including self-reported flares, primary-care visits, secondary-care visits, hospitalisation, impaired quality of life, absenteeism and impact of IBD on work productivity. When T1 SRDA was tested as a mediator of the relationship between distress (T0) and healthcare use and disease-related outcomes (T2), there were significant indirect (SRDA-mediated) pathways only for secondary care contacts, and significant direct pathways only for IBD-related quality of life and impact of IBD on productivity. For absenteeism, there was a significant SRDA-mediated pathway contributing to a 15 % increased rate in days off work due to IBD. For other secondary outcomes there were no significant effects (A&E), nonsignificant trends for indirect paths (flare frequency, flare severity) or nonsignificant trends for direct pathways (allied visits). Interestingly, FCP only showed significant positive associations with self-reported flare frequency and severity alongside allied visits in the mixed effects models. Because SRDA and FCP share substantial variance, part of the apparent FCP effect may be transmitted indirectly through SRDA and should be interpreted with caution. However, in the mediation analysis, FCP significantly predicted flare severity, secondary visits, absenteeism and hospitalisations only. This suggests that some healthcare use or disease-related outcomes, such as future absenteeism or worsening quality of life, may be more strongly driven by psychosocial processes related to distress or self-reported symptoms rather than objective inflammation.

Our finding that distress predicts SRDA, with no evidence of concomitant increases in FCP, aligns with previous studies investigating both outcomes concurrently (Sauk et al., 2023; Targownik et al., 2015). Indeed, approximately a quarter of IBD patients continue to experience symptoms while in biochemical remission (Fairbrass et al., 2020). The current longitudinal study suggests that psychological factors causally drive SRDA in the absence of measured inflammatory changes. As stated in the introduction, gastrointestinal symptoms can arise due to complex gut-brain interactions (Ge and Liu, 2022). By formally modelling bidirectional effects, we also showed that, within the 12-month timeframe studied, neither SRDA nor FCP drives future distress, implicating distress as a relevant factor for prognosis, rather than a by-product of severe disease.

Although sleep quality has previously been associated with flare risk (Kinnucan et al., 2013; Xu et al., 2024), we are the first to demonstrate that impaired sleep partially mediates the influence of distress on future SRDA. Impaired sleep may heighten visceral sensitivity (Finan et al., 2013; Nijs et al., 2018; Patel et al., 2016), amplify affective responses to gut stimuli (Finan et al., 2013; Topan et al., 2024; Whibley et al., 2019), and have pro-inflammatory effects (Irwin et al., 2016). The nonsignificant trend for a relationship between sleep and FCP in this cohort suggests that larger studies may reveal an inflammatory component. Given the potential impact on symptoms, inflammation and mental health, IBD outcomes may be improved by targeting sleep, potentially incorporating it within existing psychological interventions for distress. However, these findings must be interpreted with caution, given the reliance on self-report measures in this study which may have inflated estimates.

Furthermore, there were no significant relationships with other health behaviours with either SRDA or inflammatory disease activity, despite existing studies demonstrating relationships on subsequent disease activity (Jones et al., 2015; Lewis et al., 2021; Selinger et al., 2011). This may be due to sample size constraints, or the use of subjective, self-report measures. Future research should consider administering more extensive/granular questionnaires (e.g., for diet), collecting objective data from wearables (for sleep, exercise), or linking healthcare records (e.g., for pharmacy refills to assess adherence). Given the wide-ranging positive influences of these behaviours on health, they should not be dismissed due to minimal influence on IBD-specific inflammatory outcomes. IBD patients are at higher risk of developing obesity, metabolic syndrome, diabetes, cardiovascular disease, pain conditions, chronic fatigue, and osteoporosis, all of which tend to negatively impact disease prognosis, compound disease burden, reduce quality of life and increase cost (Adolph et al., 2024; Bähler et al., 2017; Larrosa Pardo et al., 2019; Lima et al., 2015; Martínez-Domínguez et al., 2023; Shen et al., 2024; Wu et al., 2022). Therefore, promoting healthy lifestyles in IBD may help mitigate some of the increased morbidity and costs in this population.

Our analysis of healthcare usage and disease-related outcomes extends earlier cross-sectional work, by establishing temporal precedence: even after adjusting for FCP, distress and SRDA inflate healthcare utilisation and productivity losses 6-months later. Recent research has shown that psychological interventions for distress (Evertsz et al., 2024; Lores et al., 2019; Seaton et al., 2025b), and self-management of symptoms (Moss-Morris et al., 2025) in IBD reduce healthcare usage and are cost-effective. In an era of escalating biologic expenditure and workforce losses attributable to IBD, these data strengthen the economic argument for integrating psychological care into routine management.

The key strengths of this study include the parallel collection of PROMs, FCP, and a broad array of healthcare use and disease-related outcomes that allow for a better understanding of the multifaceted impact of distress in IBD. Moreover, by incorporating health behaviours as mediators, we identified a treatable target, sleep disturbance, that is modifiable, inexpensive to treat and may influence mental health and disease-related outcomes in IBD. However, there were some key limitations: 1) The only biomarker, FCP, is limited in detecting small bowel inflammation (Cerrillo et al., 2015; Kopylov et al., 2015; Stawczyk-Eder et al., 2015), and was only feasible to be collected at baseline and 6-months. Inclusion of other biomarkers, including those more consistently associated with psychological distress (Aniwan et al., 2018; Dragasevic et al., 2024; Gao et al., 2023; Khan et al., 2013; Tang et al., 2020) at all waves would have provided a more comprehensive picture of inflammatory status. 2) Online recruitment, although opportunistic, and enabling nationwide participation, introduces some limitations. Selection bias may result in a sample that over-represents certain characteristics, for instance, help-seeking, interest in complementary medicine, or reduced trust in healthcare professionals. Relatedly, although participants underwent a screening interview to confirm diagnoses, the sample was recruited online which precluded access to medical records, thus limiting clinical validity. In terms of demographics, male sex and low education groups were significantly under-represented. The majority of the sample was white (82.1 %), however, the proportion was not dissimilar to UK 2021 population census data (81.7 %) (Office for National Statistics, 2021). Although attrition rates were low, dropouts had higher SRDA and lower education level at baseline. 3) The sample size of this study was relatively small, so findings should be interpreted cautiously. Although power was adequate for primary analyses with self-reported measures, given the noise in biomarker data, larger samples may be needed to detect mediation effects with FCP. Moreover, this study only tested isolated pathways, as the sample was not large enough to test the overall GBBO framework. Future research should perform full model testing and confirm these relationships in fully powered analyses. 4) Relatedly, this study was exploratory and did not correct for multiple statistical comparisons. The aim of this study was to collect extensive health-related outcomes to capture the multifaceted nature of health and disease impact in IBD, including five mediator variables and ten outcomes. This was likely to increase the risk of Type I error, so a formal correction may have improved robustness of the research. However, the study was explicitly hypothesis-generating and exploratory, rather than confirmatory. Breadth was prioritised, to identify outcomes and mechanisms most promising for future trials, over prematurely discarding potentially meaningful effects through conservative adjustments. This approach is generally consistent with recommendations in early-phase behavioural and mechanistic research, where more weight is placed on estimation, effect sizes and patterns across outcomes, rather than strict dichotomisation by p-values (Peeters, 2016). 5) The healthy eating assessment used to measure diet quality had low internal consistency, which may limit the interpretation of relevant analyses. This may be overly cautious as items in a measure for diet, unlike one for distress or a different psychological/behavioural construct, are not designed to measure the same thing, but to capture distinct behaviours related to an overarching concept. Future research should use more granular, accurate or reliable methods to measure diet (e.g., food diary, food frequency questionnaires), however, this study prioritised a brief tool to explore potentially relevant effects, while minimising participant burden. 6) Although we controlled for biologic and steroid use, differential effects of individual agents could not be investigated, nor could other unmeasured factors such as microbiome composition.

## 6 Conclusions

This study adds to the growing body of evidence that psychological distress influences clinical activity, healthcare usage and economic burden of IBD. Moreover, we add mechanistic detail to support trials of psychosocial interventions that demonstrate improvements in subjective disease activity, health-related costs and wider economic burden. Specifically, we identified that impaired sleep may be a key behavioural pathway which amplifies future symptom reports in IBD. Objective inflammation measured by FCP, though undoubtedly important, explains comparatively little variance in healthcare utilisation or productivity losses once psychosocial factors are considered. This research has important clinical implications, highlighting the need to 1) routinely screen for distress and impaired sleep in IBD clinics and 2) offer scalable interventions for psychological distress, potentially including a focus on improving quality of sleep which may boost the impact on both mood and self-reported disease activity.

## CRediT authorship contribution statement

**Natasha Seaton:** Writing – original draft, Supervision, Project administration, Methodology, Investigation, Formal analysis, Data curation, Conceptualization. **Joanna L. Hudson:** Writing – review & editing, Supervision, Methodology, Formal analysis, Conceptualization. **Valeria Mondelli:** Writing – review & editing, Supervision, Conceptualization. **Leah Raymond:** Project administration, Investigation, Data curation. **Pooja Schmill:** Writing – review & editing, Project administration. **Ailsa Hart:** Writing – review & editing, Validation, Methodology, Conceptualization. **Rona Moss-Morris:** Writing – review & editing, Supervision, Methodology, Conceptualization.

## Declarations

The authors declare the following financial interests/personal relationships which may be considered as potential competing interests: RMM reports personal fees from training in CBT for irritable bowel syndrome outside the submitted work. She received payment for consultancy to Mahana Therapeutics, has share options in Mahana therapeutics, and is a beneficiary of a licence agreement between Mahana therapeutics and King's College London. She has received reimbursement for travel and speaker fees from EABM, APS and BABCP conferences. AH received advisory board or lecture fees with AbbVie, Falk, J&J, Takeda, BMS, Galapogos, Lilly, GSK, Celltrion, and Pfizer. RP received a consultancy fee from Galapagos and support for travel from Celltrion Healthcare and Sun Pharma. All other authors report no competing interests.

This work was supported by the Medical Research Council (funding for NS, grant code: MR/N013700/1) and the NIHR Maudsley Biomedical Research Centre at South London and Maudsley NHS Foundation Trust and King's College London (funding for JH, VM, RMM, grant code: NIHR203318). The views expressed are those of the author(s) and not necessarily those of the NIHR or the Department of Health and Social Care.

The data that support the findings of this study are available from the corresponding author on reasonable request subject to appropriate data sharing permissions.

## Funding

This work was funded by the Medical Research Council (funding for NS and materials, grant code: MR/N013700/1) and the NIHR Maudsley Biomedical Research Centre at South London and Maudsley NHS Foundation Trust and King's College London (funding for JH, VM, RMM, grant code: NIHR203318). The views expressed are those of the author(s) and not necessarily those of the NIHR or the Department of Health and Social Care.

## Declaration of competing interest

RE: Psychological Distress Predicts Disease Activity in Inflammatory Bowel Disease: Results from the Mind-Body IBD Longitudinal Study.

## Data Availability

Data will be made available on request.
