# Supplementary material for: Psychological distress predicts disease activity in inflammatory bowel disease: Results from the mind-body IBD longitudinal study
Source: Brain Behav Immun Health. 2025 Dec 3;51:101147. doi: 10.1016/j.bbih.2025.101147 (PMC12765445; doi:10.1016/j.bbih.2025.101147)
Supplement: Multimedia component 1 [file mmc1.docx]

**Supplementary Material**

**Supplement 1. Flow of participants through the study**


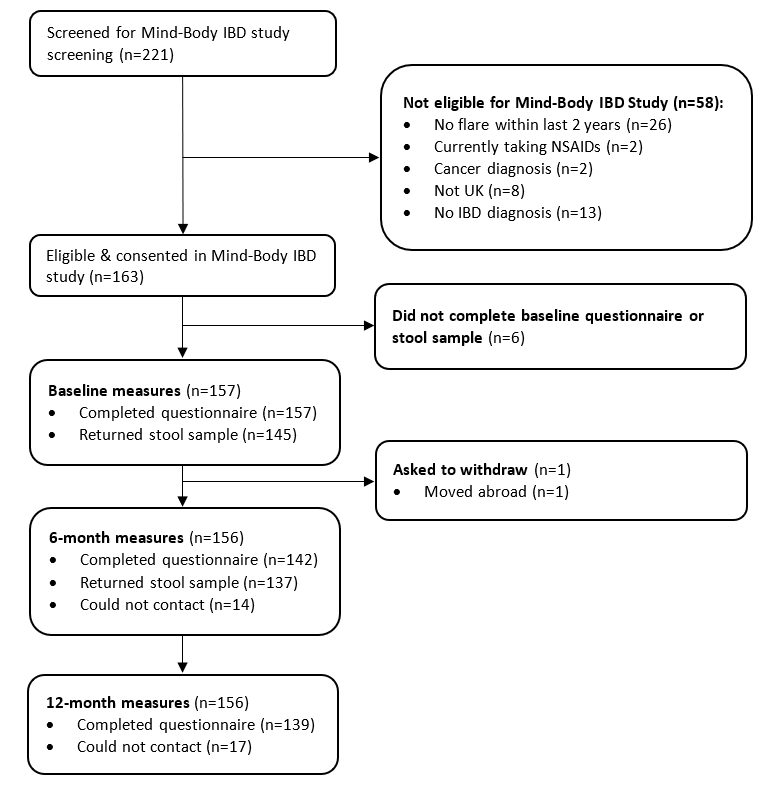


**Supplement 2. Coding of demographic and clinical covariate variables**

For ethnicity, education and employment, categories with small sample sizes were collapsed so they could still be included as meaningful covariates, as shown in the table below. Ethnicity options were aligned with categories used by the Office of National Statistics and the UK Census.

| Covariate labels | | Questionnaire response options |
| --- | --- | --- |
| Ethnicity | |  |
|  | White | English, Welsh, Scottish, Northern Irish, British; Irish; Gypsy or Irish traveller; Any other white background |
|  | Mixed | White and Black Caribbean; White and Black African; White and Asian; Any other mixed or multiple ethnic background |
|  | Asian or Asian British | Chinese; Indian; Bangladeshi; Pakistani; Any other Asian background |
|  | Black or Black British | African; Caribbean; Any other Black, African or Caribbean background |
|  | Other ethnic group | Arab; Any other ethnic group |
| Education | |  |
|  | None | None |
|  | School | GCSEs; A-levels |
|  | Undergraduate | Undergraduate |
|  | Postgraduate | Postgraduate (excluding PhD and Doctorate); Doctoral level |
| Employment | |  |
|  | Employed | Employed |
|  | Unemployed | Not working and no benefits, long-term sick or disabled, actively seeking work unpaid voluntary work |
|  | Student | Student |
|  | Retired | Retired, homemaker |

For inferential statistical analysis, those with indeterminate colitis or IBD-U were group with ulcerative colitis, in line with other studies and instructions from the patient-reported outcome measure for disease activity.^1^

BMI was calculated by dividing adult's weight in kilograms by their height in metres squared. Standard BMI cutoffs were used to classify weight categories: underweight is below 18.5, healthy weight is 18.5–24.9, overweight is 25.0–29.9, and obese is 30.0 or higher.

**Supplement 3. Demographic and clinical differences between dropped out and retained participants.**

| Variable | | Retained | *Dropouts* | Statistical test | *p* value |
| --- | --- | --- | --- | --- | --- |
|  |  | M(SD), n(%) | M(SD), n(%) |  |  |
| Demographic characteristics | |  |  |  |  |
| Age | | 36.17 (11.72) | 31.55 (8.70) | *t*=1.28 | .202 |
| Female gender | | 109 (74.7%) | 7 (63.6%) | Χ^2^=0.64 | .422 |
| Minority ethnic group | | 27 (18.5%) | 1 (9.09%) | Χ^2^=0.62 | .432 |
| Index of Multiple Deprivation Decile^$^ | | 0.54 (0.28) | 0.48 (0.33) | *t*=0.69 | .494 |
| Unemployed status | | 11 (7.5%) | 1 (9.1%) | Χ^2^=0.04 | .851 |
| Education | |  |  | Χ^2^=8.71 | .033 |
|  | None | 1 (0.7%) | 0 (0.0%) |  |  |
|  | School | 27 (18.5%) | 5 (54.6%) |  |  |
|  | Undergraduate | 66 (45.2%) | 4 (36.36%) |  |  |
|  | Postgraduate | 52 (35.6%) | 1 (9.1%) |  |  |
| Clinical characteristics | |  |  |  |  |
| Crohn’s Disease diagnosis | | 50 (34.3%) | 2 (18.18%) | Χ^2^=1.19 | .275 |
| Body Mass Index | | 24.97 (5.36) | 24.74 (7.39) | *t*=0.13 | .894 |
| Years since diagnosis (min= 0, max= 40) | |  |  |  |  |
| Stoma presence | | 8 (5.5%) | 1 (9.1%) | Χ^2^=0.25 | .619 |
| FCP (log transformed) | | 4.09 (1.79) | 3.78 (1.55) | *t*=0.424 | .672 |
| Anxiety (GAD-7) | | 8.43 (5.46) | 10.73 (5.00) | *t*=-1.35 | .178 |
| Depression (PHQ-9) | | 8.55 (5.89) | 11.09 (4.68) | *t*=-1.39 | .165 |
| Distress (PHQ-ADS) | | 16.99 (10.44) | 21.82 (8.85) | *t*=-1.49 | .137 |
| Disease activity PROM | | 0.05 (0.96) | 0.82 (1.34) | *t*=-2.39 | .018 |

**Supplement 4. Relationships of demographic and clinical variables with distress, self-reported disease activity and faecal calprotectin.**

| Demographic characteristics | | | Distress | | Self-reported disease activity | | Faecal calprotectin | |
| --- | --- | --- | --- | --- | --- | --- | --- | --- |
|  | | | *β* | 95%CI | *β* | 95%CI | *β* | 95%CI |
| Age | | | 0.002 | -0.15, 0.16 | -0.012 | -0.18, 0.15 | 0.033 | -0.13, 0.20 |
| Female gender | | | 0.192* | 0.04, 0.34 | 0.016 | -0.15, 0.18 | -0.197* | -0.36, -0.03 |
| Ethnicity (white=reference) | | |  |  |  |  |  |  |
|  | Mixed/multiple ethnic groups | | -0.046 | -0.20, 0.11 | -0.015 | -0.17, 0.14 | 0.201* | 0.04, 0.36 |
|  | Asian or Asian British | | -0.007 | -0.16, 0.15 | 0.059 | -0.10, 0.22 | 0.089 | -0.09, 0.27 |
|  | Black or Black British | | 0.170* | 0.02, 0.32 | 0.343** | 0.12, 0.56 | -0.018 | -0.18, 0.14 |
|  | Arab | | 0.119 | -0.05, 0.26 | -0.040 | -0.20, 0.12 | -0.027 | -0.19, 0.13 |
| Index of Multiple Deprivation Decile^$^ | | | -0.076 | -0.23, 0.08 | -0.207* | -0.37, -0.05 | 0.016 | -0.15, 0.19 |
| Employment Status (employed=reference) | | |  |  |  |  |  |  |
|  | Unemployed | | 0.220** | 0.07, 0.37 | 0.218** | 0.06, 0.37 | 0.021 | -0.14, 0.18 |
|  | Retired | | -0.030 | -0.18, 0.12 | -0.039 | -0.20, 0.12 | 0.169* | 0.00, 0.34 |
|  | Student | | 0.021 | -0.13, 0.17 | -0.167* | -0.34, 0.00 | -0.042 | -0.20. 0.12 |
| Education (school=reference) | | |  |  |  |  |  |  |
|  | Undergraduate | | 0.011 | -0.20, 0.22 | -0.198 | -0.41, 0.01 | -0.173 | -0.40, 0.05 |
|  | Postgraduate | | 0.050 | -0.16, 0.26 | -0.056 | -0.27, 0.15 | -0.190 | -0.41, 0.03 |
|  | None | | 0.108 | -0.05, 0.27 | 0.109 | -0.05, 0.27 | -0.121 | -0.28, 0.04 |
| Clinical characteristics | | |  |  |  |  |  |  |
| Diagnosis (Crohn’s Disease=reference) | | |  |  |  |  |  |  |
|  | Ulcerative Colitis | | -0.097 | -0.26, 0.07 | 0.021 | -0.15, 0.19 | 0.179* | 0.00, 0.36 |
|  | Indeterminate | | -0.043 | -0.21, 0.12 | -0.061 | -0.25, 0.13 | 0.065 | -0.10, 0.23 |
|  | Unsure | | 0.036 | -0.12, 0.19 | -0.043 | -0.21, 0.12 | 0.028 | -0.20, 0.26 |
| Body Mass Index (healthy weight=reference) | | |  |  |  |  |  |  |
|  | Overweight | | 0.258** | 0.10, 0.41 | 0.237** | 0.08, 0.39 | 0.049 | -0.12, 0.22 |
|  | Underweight | | 0.057 | -0.10, 0.21 | 0.202* | 0.04, 0.37 | 0.080 | -0.11, 0.27 |
| Years since diagnosis (min= 0, max= 40) | | | 0.020 | -0.14, 0.18 | 0.135 | -0.02, 0.29 | 0.058 | -0.11, 0.22 |
| Medications | | |  |  |  |  |  |  |
|  | Aminosalicylates/5-ASA | | -0.082 | -0.28, 0.12 | 0.144 | -0.06, 0.34 | 0.149 | -0.06, 0.36 |
|  | Immunomodulators | | 0.115 | -0.05, 0.27 | 0.069 | -0.09, 0.23 | -0.013 | -0.18, 0.15 |
|  | Biologics | | -0.038 | -0.24, 0.17 | -0.015 | -0.22, 0.19 | -0.065 | -0.27, 0.14 |
|  | Steroids | | 0.181* | 0.02, 0.34 | 0.312*** | 0.15, 0.47 | 0.270** | 0.11, 0.43 |
|  | No IBD medication | | -0.006 | -0.21, 0.20 | 0.026 | -0.18, 0.23 | -0.008 | -0.23, 0.21 |
| Stoma presence | | | 0.073 | -0.08, 0.23 | -0.118 | -0.28, 0.04 | -0.198* | -0.37, -0.03 |
| FCP (linear) | | | -0.020 | -0.18, 0.14 | 0.273** | 0.12, 0.43 |  |  |
| FCP (categorical, remission=reference) | | |  |  |  |  |  |  |
|  | Borderline | | 0.146 | -0.01, 0.31 | 0.123 | -0.04, 0.29 |  |  |
|  | Active disease | | -0.018 | -0.18, 0.14 | 0.182* | 0.02, 0.35 |  |  |
|  | Missing | | 0.101 | -0.06, 0.26 | 0.126 | -0.04, 0.29 |  |  |
| Anxiety (GAD-7) | | | 0.918*** | 0.85, 0.98 | 0.373*** | 0.22, 0.53 | -0.080 | -0.25, 0.09 |
| Depression (PHQ-9) | | | 0.910*** | 0.85, 0.97 | 0.403*** | 0.25, 0.55 | 0.038 | -0.13, 0.21 |
| Distress (PHQ-ADS) | | |  |  | 0.426*** | 0.28, 0.58 | -0.021 | -0.19, 0.15 |
| Disease activity PROM | | | 0.404*** | 0.26, 0.55 |  |  | 0.291** | 0.13, 0.46 |
| Health behaviours | | |  |  |  |  |  |  |
| Impaired sleep quality (PSQI) | | | 0.645*** | 0.52, 0.77 | 0.379*** | 0.22, 0.54 | 0.058 | -0.12, 0.23 |
| Diet quality | | | -0.194* | -0.35, -0.04 | -0.106 | -0.27, 0.06 | -0.233** | -0.40, -0.07 |
| Exercise (IPAQ total) | | | -0.252** | -0.41, -0.10 | -0.160 | -0.31, 0.01 | -0.203* | -0.38, -0.03 |
|  | | Vigorous (minutes) | -0.194* | -0.35, -0.04 | -0.055 | -0.22, 0.11 | -0.160 | -0.34, 0.02 |
|  | | Moderate (minutes) | -0.066 | -0.24, 0.11 | 0.075 | -0.11, 0.25 | -0.009 | -0.21, 0.18 |
|  | | Walking (minutes) | -0.108 | -0.27, 0.06 | -0.125 | -0.29, 0.04 | -0.093 | -0.27, 0.08 |
| Adherent (MARS=25) | | | -0.050 | -0.21, 0.11 | -0.190* | -0.35, -0.03 | 0.015 | -0.15, 0.18 |
| Current smoker | | | 0.146 | -0.01, 0.30 | 0.005 | -0.15, 0.17 | -0.086 | -0.28, 0.11 |

$ deprivation was computed using postcodes in national registries, deprivation data was missing for 3 postcodes. FCP=faecal calprotectin, GAD-7=Generalised Anxiety Disorder Scale, IBD=Inflammatory Bowel Disease, IPAQ=International Physical Activity Questionnaire, IQR=interquartile range, MARS=Medication Adherence Rating Scale, PHQ-9 Patient Health Questionnaire, PHQ-ADS=Patient Health Questionnaire Anxiety and Depression Scale, PROM=patient reported outcome measure, PSQI=Pittsburgh Sleep Quality Index, UC=ulcerative colitis.

**Supplement 5. Results of cross-lagged panel model for the reciprocal associations between psychological distress and self-reported disease activity (SRDA) over a 12-month period**

|  | *β* | SE | *p* | 95% Confidence Interval | |
| --- | --- | --- | --- | --- | --- |
|  |  |  |  | lower bound | upper bound |
| **Psychological Distress T1** |  |  |  |  |  |
| Psychological Distress T0 | 0.709 | 0.07 | <.001 | 0.57 | 0.85 |
| SRDA T0 | -0.037 | 0.07 | .617 | -0.18 | 0.11 |
|  |  |  |  |  |  |
| **SRDA T1** |  |  |  |  |  |
| Psychological Distress T0 | 0.159 | 0.07 | .030 | 0.02 | 0.30 |
| SRDA T0 | 0.448 | 0.08 | <.001 | 0.30 | 0.60 |
|  |  |  |  |  |  |
| **Psychological Distress T2** |  |  |  |  |  |
| Psychological Distress T1 | 0.611 | 0.07 | <.001 | 0.47 | 0.75 |
| SRDA T1 | 0.043 | 0.08 | .577 | -0.11 | 0.20 |
|  |  |  |  |  |  |
| **SRDA T2** |  |  |  |  |  |
| Psychological Distress T1 | 0.023 | 0.00 | .797 | -0.15 | 0.20 |
| SRDA T1 | 0.450 | 0.10 | <.001 | 0.26 | 0.64 |
|  |  |  |  |  |  |
| **Covariance** |  |  |  |  |  |
| T0 | 0.409 | 0.09 | <.001 | 0.24 | 0.58 |
| T1 | 0.218 | 0.05 | <.001 | 0.11 | 0.32 |
| T2 | 0.313 | 0.07 | <.001 | 0.18 | 0.45 |

*Note***.** SRDA=self-reported disease activity.

**Supplement 6. Results of cross-lagged panel model for the reciprocal associations between psychological distress and faecal calprotectin (FCP) over a 6-month period**

|  |  |  |  | 95% Confidence Interval | |
| --- | --- | --- | --- | --- | --- |
|  | β | SE | p value | lower bound | upper bound |
| **Psychological Distress T1** | |  |  |  |  |
| Psychological Distress T0 | 0.675 | 0.07 | <.001 | 0.55 | 0.80 |
| FCP T0 | -0.030 | 0.06 | .643 | -0.16 | 0.10 |
|  |  |  |  |  |  |
| **FCP T1** |  |  |  |  |  |
| Psychological Distress T0 | 0.037 | 0.07 | .610 | -0.11 | 0.18 |
| FCP T0 | 0.535 | 0.07 | <.001 | 0.40 | 0.67 |
|  |  |  |  |  |  |
| **Covariance** |  |  |  |  |  |
| T0 | -0.007 | 0.08 | .934 | -0.17 | 0.15 |
| T1 | 0.129 | 0.05 | .019 | 0.02 | 0.24 |

*Note*. FCP=faecal calprotectin.

**Supplement 7. Sensitivity analysis for modelling health behaviours as mediators of the effect of distress on disease activity while controlling for relevant covariates**

These sensitivity analyses were run controlling for age, gender, overweight BMI, biologics use, and steroids use.

|  |  |  | Self-reported disease activity | | |  | Faecal Calprotectin | | |
| --- | --- | --- | --- | --- | --- | --- | --- | --- | --- |
|  |  |  | β (SE) | p | 95%CIs |  | β (SE) | p | 95%CIs |
| Total effect | | Distress→DA | 0.112 (0.00) | .194 | -0.06, 0.28 |  | 0.033 (0.08) | .663 | -0.12, 0.18 |
|  | |  |  |  |  |  |  |  |  |
| Sleep | |  |  |  |  |  |  |  |  |
|  | Path a | Distress→Sleep | 0.420 (0.08) | <.001 | 0.25, 0.59 |  | 0.019 (0.08) | .806 | -0.13, 0.17 |
|  | Path b | Sleep→DA | 0.182 (0.09) | .048 | 0.00, 0.36 |  | 0.214 (0.11) | .048 | -0.08, 0.29 |
|  | Indirect | Distress→Sleep→ DA | 0.076 (0.04) | .068 | -0.01, 0.16 |  | 0.004 (0.02) | .808 | -0.03, 0.04 |
|  | Direct | Distress→DA* | 0.025 (0.09) | .795 | -0.16, 0.21 |  | 0.107 (0.09) | .252 | -0.08, 0.29 |
| Diet | |  |  |  |  |  |  |  |  |
|  | Path a | Distress→Diet | -0.307 (0.10) | .001 | -0.50, -0.12 |  | -0.138 (0.08) | .070 | -0.29, 0.01 |
|  | Path b | Diet → DA | -0.048 (0.08) | .555 | -0.21, 0.11 |  | -0.205 (0.09) | .019 | -0.38, -0.03 |
|  | Indirect | Distress→ Diet → DA | 0.015 (0.03) | .562 | -0.04, 0.07 |  | 0.028 (0.02) | .152 | -0.01, 0.07 |
|  | Direct | Distress→DA* | 0.098 (0.08) | .266 | -0.07, 0.11 |  | -0.012 (0.08) | .877 | -0.16, 0.14 |
| Exercise | |  |  |  |  |  |  |  |  |
|  | Path a | Distress→ Exercise | -0.289 (0.08) | .001 | -0.45, -0.12 |  | -0.211 (0.08) | .005 | -0.36, -0.06 |
|  | Path b | Exercise→ DA | 0.082 (0.09) | .376 | -0.04, 0.32 |  | -0.011 (0.09) | .904 | -0.19, 0.16 |
|  | Indirect | Distress→Exercise→ DA | -0.024 (0.03) | .391 | -0.08, 0.03 |  | 0.002 (0.02) | .904 | -0.03, 0.04 |
|  | Direct | Distress→ DA* | 0.138 (0.09) | .129 | -0.01, 0.26 |  | 0.059 (0.08) | .452 | -0.09, 0.21 |
| Adherence | |  |  |  |  |  |  |  |  |
|  | Path a | Distress→ Adherence | -0.074 (0.10) | .439 | -0.26, 0.11 |  | -0.024 (0.07) | .740 | -0.17, 0.12 |
|  | Path b | Adherence → DA | 0.048 (0.08) | .561 | -0.11, 0.21 |  | -0.007 (0.09) | .940 | -0.18, 0.19 |
|  | Indirect | Distress→Adherence→DA | -0.004(0.01) | .641 | -0.02, 0.01 |  | 0.000 (0.00) | .942 | -0.00, 0.00 |
|  | Direct | Distress→DA* | 0.117 (0.09) | .176 | -0.05, 0.29 |  | -0.010 (0.08) | .891 | -0.16, 0.14 |
| Smoking | |  |  |  |  |  |  |  |  |
|  | Path a | Distress→ Smoking | 0.116 (0.13) | .363 | -0.13, 0.37 |  | 0.077 (0.12) | .522 | -0.16, 0.31 |
|  | Path b | Smoking → DA | -0.005 (0.13) | .932 | -0.12, 0.11 |  | 0.051 (0.06) | .369 | -0.06, 0.16 |
|  | Indirect | Distress→Smoking→DA | -0.001 (0.01) | .932 | -0.01, 0.01 |  | 0.004 (0.01) | .602 | -0.01, 0.02 |
|  | Direct | Distress→DA* | 0.113 (0.09) | .192 | -0.06, 0.28 |  | 0.051 (0.06) | .697 | -0.12, 0.18 |

**Supplement 8. Sensitivity analysis for mixed models of psychological distress or SRDA predicting secondary healthcare usage and disease-related outcomes, statistically controlling for baseline values and FCP.**

These sensitivity analyses were run controlling for age, gender, overweight BMI, biologic use and steroid use.

|  |  | Faecal Calprotectin | | | Psychological Distress | | | Self-reported disease activity | | |
| --- | --- | --- | --- | --- | --- | --- | --- | --- | --- | --- |
|  |  | IRR/β/OR | p | 95%CIs | IRR/β/OR | p | 95%CIs | IRR/β/OR | p | 95%CIs |
| Flares total (IRR) | | |  |  |  |  |  |  |  |  |
|  | Model 1 | 1.314 (0.13) | .007 | 1.08, 1.60 | 1.288 (0.13) | .012 | 1.06, 1.57 |  |  |  |
|  | Model 2 | 1.218 (0.12) | .046 | 1.00, 1.48 |  |  |  | 1.46 (0.13) | <.001 | 1.23, 1.74 |
| Flare severity (OR) | | |  |  |  |  |  |  |  |  |
|  | Model 1 | 2.267 (0.51) | <.001 | 1.45, 3.53 | 1.821 (0.41) | .008 | 1.17, 2.84 |  |  |  |
|  | Model 2 | 1.964 (0.42) | .002 | 1.29, 2.99 |  |  |  | 2.453 (0.58) | <.001 | 1.55, 3.89 |
| Primary visits (IRR) | | |  |  |  |  |  |  |  |  |
|  | Model 1 | 0.926 (0.05) | .169 | 0.83, 1.03 | 1.097 (0.06) | .078 | 0.99, 1.22 |  |  |  |
|  | Model 2 | 0.885 (0.05) | .037 | 0.79, 0.99 |  |  |  | 1.100 (0.06) | .080 | 0.99, 1.22 |
| Secondary visits (IRR) | | |  |  |  |  |  |  |  |  |
|  | Model 1 | 1.078 (0.07) | .224 | 0.96, 1.22 | 1.037 (0.07) | .579 | 0.91, 1.18 |  |  |  |
|  | Model 2 | 1.032 (0.07) | .622 | 0.91, 1.17 |  |  |  | 1.185 (0.07) | .005 | 1.05, 1.33 |
| Allied visits (IRR) | | |  |  |  |  |  |  |  |  |
|  | Model 1 | 1.116 (0.12) | .293 | 0.91, 1.37 | 1.215 (0.11) | .036 | 1.01, 1.46 |  |  |  |
|  | Model 2 | 1.100 (0.12) | .380 | 0.89, 1.36 |  |  |  | 1.019 (0.10) | .853 | 0.84, 1.24 |
| Absenteeism (IRR) | | |  |  |  |  |  |  |  |  |
|  | Model 1 | 1.031 (0.12) | .789 | 0.82, 1.29 | 1.815 (0.22) | <.001 | 1.43, 2.30 |  |  |  |
|  | Model 2 | 0.971 (0.12) | .804 | 0.77, 1.23 |  |  |  | 1.534 (0.19) | .001 | 1.20, 1.96 |
| Hospitalisations (IRR) | | |  |  |  |  |  |  |  |  |
|  | Model 1 | 1.443 (0.38) | .159 | 0.87, 2.40 | 1.573 (0.38) | .060 | 0.98, 2.52 |  |  |  |
|  | Model 2 | 1.144 (0.33) | .638 | 0.65, 2.01 |  |  |  | 1.510 (0.35) | .075 | 0.96, 2.38 |
| Productivity impact (β) | | |  |  |  |  |  |  |  |  |
|  | Model 1 | 0.056 (0.06) | .339 | -0.06, 0.17 | 0.215 (0.07) | .001 | 0.08, 0.35 |  |  |  |
|  | Model 2 | 0.045 (0.06) | .470 | -0.08, 0.17 |  |  |  | 0.130 (0.07) | .053 | 0.00, 0.26 |
| IBD-QoL (β) | |  |  |  |  |  |  |  |  |  |
|  | Model 1 | 0.028 (0.04) | .507 | -0.05, 0.11 | -0.119 (0.05) | .027 | -0.22, -0.01 |  |  |  |
|  | Model 2 | 0.049 (0.04) | .271 | -0.04, 0.14 |  |  |  | -0.109 (0.05) | .029 | -0.21, -0.01 |
| A&E visit (OR) | |  |  |  |  |  |  |  |  |  |
|  | Model 1 | 1.475 (0.45) | .199 | 0.82, 2.67 | 0.987 (0.03) | .659 | 0.93, 1.05 |  |  |  |
|  | Model 2 | 1.230 (0.36) | .479 | 0.69, 2.18 |  |  |  | 1.254 (0.34) | .408 | 0.73, 2.14 |

*Note*. For the following analyses, the model did not converge with all covariates included, so age was omitted: Flare severity Model 1, Flare severity Model 2, Primary visits Model 1, Hospitalisations Model 1. A&E=accident and emergency, FCP=faecal calprotectin IBD=inflammatory bowel disease, IRR=incident risk ratio, OR=odds ratio. QoL=quality of life
